# Texture Analysis Using Semiquantitative Kinetic Parameter Maps from DCE-MRI: Preoperative Prediction of HER2 Status in Breast Cancer

**DOI:** 10.3389/fonc.2021.675160

**Published:** 2021-06-08

**Authors:** Lirong Song, Chunli Li, Jiandong Yin

**Affiliations:** ^1^ Department of Radiology, Shengjing Hospital of China Medical University, Shenyang, China; ^2^ Department of Biomedical Engineering, School of Fundamental Sciences, China Medical University, Shenyang, China

**Keywords:** breast cancer, HER2, dynamic contrast-enhanced magnetic resonance imaging, texture analysis, semiquantitative kinetic parameter map

## Abstract

**Objective:**

To evaluate whether texture features derived from semiquantitative kinetic parameter maps based on breast dynamic contrast-enhanced magnetic resonance imaging (DCE-MRI) can determine human epidermal growth factor receptor 2 (HER2) status of patients with breast cancer.

**Materials and Methods:**

This study included 102 patients with histologically confirmed breast cancer, all of whom underwent preoperative breast DCE-MRI and were enrolled retrospectively. This cohort included 48 HER2-positive cases and 54 HER2-negative cases. Seven semiquantitative kinetic parameter maps were calculated on the lesion area. A total of 55 texture features were extracted from each kinetic parameter map. Patients were randomly divided into training (n = 72) and test (n = 30) sets. The least absolute shrinkage and selection operator (LASSO) was used to select features in the training set, and then, multivariate logistic regression analysis was conducted to establish the prediction models. The classification performance was evaluated by receiver operating characteristic (ROC) analysis.

**Results:**

Among the seven prediction models, the model with features extracted from the early signal enhancement ratio (ESER) map yielded an area under the ROC curve (AUC) of 0.83 in the training set (sensitivity of 70.59%, specificity of 92.11%, and accuracy of 81.94%), and the highest AUC of 0.83 in the test set (sensitivity of 57.14%, specificity of 100.00%, and accuracy of 80.00%). The model with features extracted from the slope of signal intensity (SI_slope_) map yielded the highest AUC of 0.92 in the training set (sensitivity of 82.35%, specificity of 97.37%, and accuracy of 90.28%), and an AUC of 0.79 in the test set (sensitivity of 92.86%, specificity of 68.75%, and accuracy of 80.00%).

**Conclusions:**

Texture features derived from kinetic parameter maps, calculated based on breast DCE-MRI, have the potential to be used as imaging biomarkers to distinguish HER2-positive and HER2-negative breast cancer.

## Introduction

Breast cancer is one of the most common cancers in women, and breast cancer alone accounts for 30% of new cancer cases in females (1). The status of human epidermal growth factor receptor 2 (HER2) is a biological factor that influences breast cancer survival. The 5-year relative survival rate has increased to 91% largely due to improvements in treatment, such as aromatase inhibitors for hormone receptor-positive tumors and trastuzumab for HER2-positive tumors. Of patients with hormone receptor-positive tumors, 81% receive hormonal therapy (2). With the development of new therapeutic drugs, better responses are seen with more specific pharmaceutical treatment options based on different molecular markers (3, 4). Therefore, it is very important to accurately identify HER2 status to individualize treatment. At present, HER2 amplification status is determined by immunohistochemistry (IHC); tumors are considered to be HER2-positive if the IHC analysis is scored as 3, whereas tumors are considered to be HER2-negative if scored as 0 or 1. For tumors with IHC scores of 2, further analysis by fluorescence *in situ* hybridization (FISH) is needed to detect the amplification status of the HER2 gene. However, these methods require invasive biopsies, and are also subject to sampling errors due to intratumoral heterogeneity (5). Moreover, FISH examination is costly and time-consuming. Thus, it would be clinically beneficial to develop a cost- and time-effective, accurate, noninvasive method to detect HER2 status.

Breast dynamic contrast-enhanced magnetic resonance imaging (DCE-MRI) is the most widely used and clinically proven imaging technique in breast cancer, and it has high sensitivity for detecting breast lesions. It provides anatomical information as well as hemodynamic information of the tumor with a high spatial resolution (6). Previous studies on breast DCE-MRI have indicated that morphological and kinetic characteristics were associated with benign and malignant tumors, response to neoadjuvant chemotherapy, and histopathological factors of breast cancer (7–11).

Texture analysis has been widely applied to characterize the spatial distribution of gray level intensities in images, capturing image patterns which are usually unrecognizable or unresolved by the human eye (12–14). This approach aims to extract high-throughput information to characterize image heterogeneity in specific target regions (15, 16). The most commonly used texture features can be layered by the statistical order of the voxel information encoded within the target regions, including first-order (also called histogram), second-order (gray level co-occurrence matrix and run-length matrix), and high-order (structural and transformed) texture features, proving to be helpful in assessing tumors. Earlier studies on rectal cancer revealed that texture features were useful for prediction of pathological complete response after neoadjuvant chemotherapy (17–19). Moreover, histogram features have been shown to be useful in evaluating tumor heterogeneity in glioma and cervical cancers (20, 21).

In previous studies, texture features derived from mammography and multidetector computed tomography images have been applied and shown to potentially identify HER2 status in patients with breast cancer (22, 23). However, DCE-MRI is recognized as the most common and effective method in breast cancer imaging. Montemurro et al. (10) showed that Fischer’s score, which included three morphological, two functional, and five DCE-MRI features, was inversely associated with HER2-overexpression. Another study demonstrated that texture features from DCE-MRI were predictive of HER2 status (24). Semiquantitative kinetic parameter maps provide a technique for leveraging the pre- and post-contrast acquisitions, and can reflect kinetic information for breast cancer. A recent study demonstrated that the model based on texture features from semiquantitative kinetic parameter maps was able to discriminate sentinel lymph node status (25). To the best of our knowledge, no previous study has investigated the association between HER2 status in breast cancer and texture features extracted from semiquantitative kinetic parameter maps calculated from breast DCE-MRI.

Thus, the aim of this study was to evaluate whether features derived from semiquantitative kinetic parameter maps could be used to identify HER2 status in patients with breast cancer.

## Materials and Methods

### Study Population

This retrospective study was approved by our institutional review board (NO.2019PS175K) and the requirement for informed consent was waived. From January 2019 to January 2020, female patients with histologically confirmed breast cancer who underwent breast DCE-MRI were reviewed with our picture archiving and communication system (PACS). The inclusion criteria were as follows: (1) visible breast lesion on DCE-MRI; (2) histologically confirmed breast cancer; and (3) exact HER2 amplification status determined by IHC/FISH examination. The exclusion criteria were: (1) patients who underwent a biopsy before MRI examination; (2) patients who received neoadjuvant chemotherapy before MRI; and (3) insufficient MRI quality due to obvious motion artifacts.

**Figure 1:**
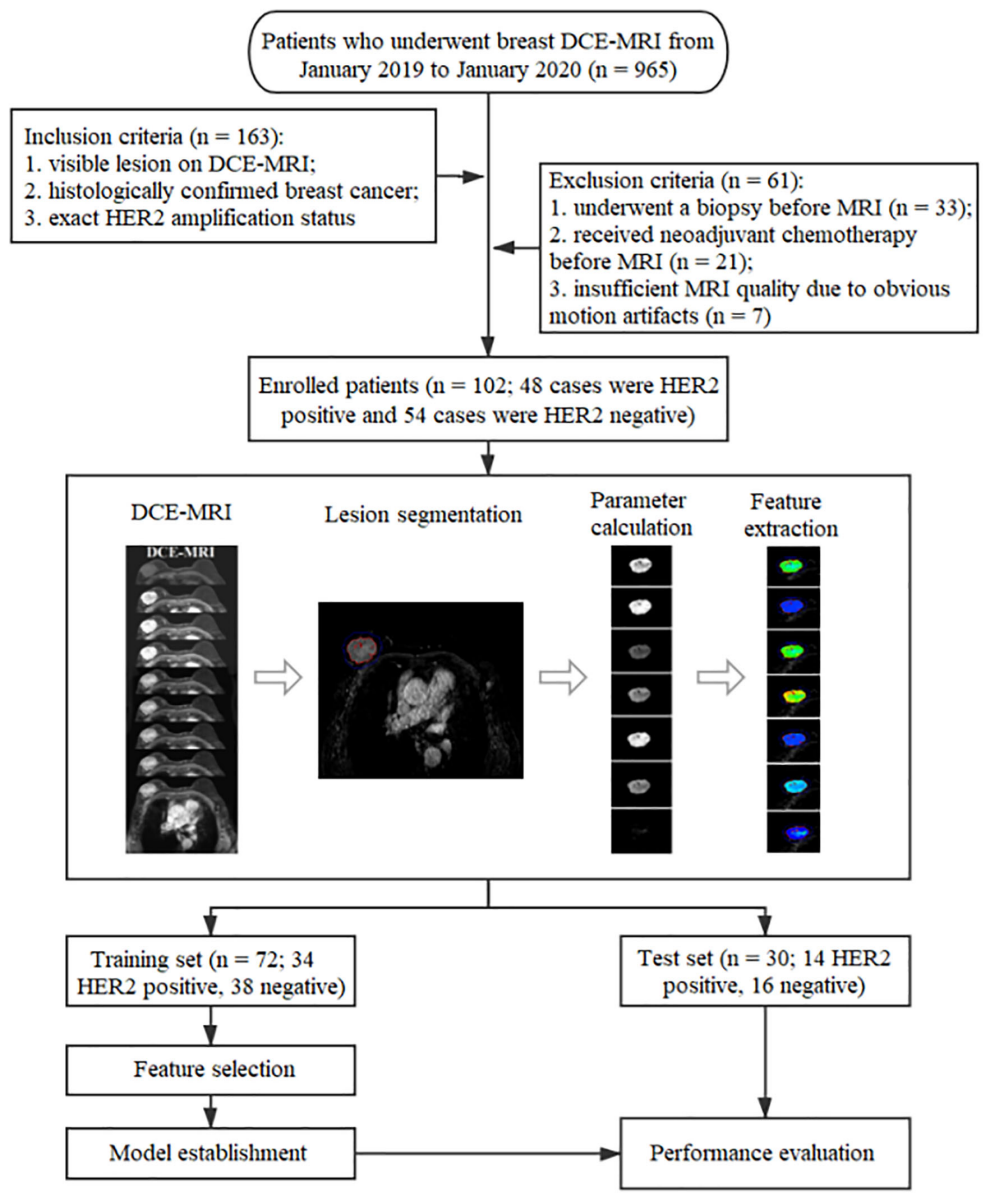
The workflow of this study.

Finally, a total of 102 patients were enrolled in this study retrospectively. Of these patients, 48 were HER2-positive and 54 were HER2-negative. The clinical characteristics collected using the PACS included age, maximum tumor diameter, estrogen receptor status, progesterone receptor status, Ki-67 status, histological grade, and histological type. Patients were randomly divided into a training set (n = 72, 34 HER2-positive and 38 HER2-negative) and a test set (n = 30, 14 HER2-positive and 16 HER2-negative) at a proportion of 70% and 30%, respectively. **Figure 1** shows the workflow of this study.

### MRI Acquisition

All patients received a pretreatment breast DCE-MRI at our institution using a 3.0 Tesla MR scanner (Ingenia, Philips Medical System, Best, Netherlands) equipped with a dedicated 7-channel bilateral breast coil with patient in a prone position. First, an axial fat-saturated T1-weighted precontrast scan based on the VIBRANT-VX technique was acquired. Then, eight axial contrast-enhanced fat-saturated T1-weighted scans were acquired after the intravenous bolus injection of a contrast agent (Magnevist, Bayer Healthcare Pharmaceuticals, Berlin, Germany) with a dose of 0.15 mmol per kg body weight. The imaging parameters were as follow: repetition time, 4.14 ms; echo time, 2.10 ms; flip angle, 12°; slice thickness, 2.00 mm; spacing between slices, 1.00 mm; field of view, 340 × 340 mm^2^; matrix, 380 × 380. Eight subtraction sequences were obtained by subtracting the precontrast scan from each of the eight postcontrast scans.

### Image Processing and Semiquantitative Kinetic Parameter Calculation

Two breast radiologists, each with over 6 years of experience, were blinded to HER2 status of patients and invited to help review the images. Slices with the maximum tumor diameter were chosen in consensus. The third-phase subtraction image, the eight phases of postcontrast images, and the precontrast image of the slice were downloaded and used for subsequent processing.

The lesion area was first delineated automatically on the third-phase subtraction image using an in-house software programmed with MATLAB 2018a (Mathworks, Natick, MA, USA). Seven semiquantitative kinetic parameter maps were calculated on the lesion area, respectively. The seven kinetic parameters included the initial percentage of enhancement (E_initial_), the percentage of peak enhancement (E_peak_), the early signal enhancement ratio (ESER), the maximum slope of increase (MSI), the second enhancement percentage (SEP), the signal enhancement ratio (SER), and the slope of signal intensity (SI_slope_). The calculation formulas of the parameters are as follows:

(1) $$E_{initial}=(SI_{1}-SI_{0})/SI_{0}\times 100\%$$

where SI_1_ and SI_0_ represent the signal intensities of the first postcontrast image and the precontrast image, respectively.

(2) $$E_{peak}=(SI_{peak}-SI_{0})/SI_{0}\times 100\%$$

where SI_peak_ represents the peak signal intensity value of the contrast enhancement.

(3) $$ESER=(SI_{1}-SI_{0})/(SI_{2}-SI_{0})\times 100\%$$

where SI_2_ represents the signal intensity at the second postcontrast time point.

(4) $$MSI=\max(SI_{i+1}-SI_{i})$$

where SI_i_ and SI_i+1_ stand for the signal intensity of a certain phase and the following phase respectively, with i ranges from 0 to 7.

(5) $$SEP=(SI_{2}-SI_{0})/SI_{0}\times 100\%$$

(6) $$SER=(SI_{peak}-SI_{0})/(SI_{8}-SI_{0})\times 100\%$$

where SI_8_ is the signal intensity at the eighth postcontrast time point.

(7) $$SI_{slope}=[(SI_{8}-SI_{mean})/SI_{mean}]\times 100\%$$

where SI_mean_ is the mean value of the signal intensity at the first two postcontrast time points.

### Texture Feature Extraction

All texture feature extraction was performed using an in-house software developed in MATLAB 2018a. Fifty-five texture features were derived from each kinetic parameter map, including histogram features, gray level co-occurrence matrix (GLCM) features, gray level run-length matrix (GRLM) features, and discrete wavelet transformation (DWT) features. The details of these features are provided in **Table 1**. GLCM parameters were calculated from four GLCMs corresponding to a distance of one pixel and four angles (0°, 45°, 90°, 135°), and the mean value of each feature over the four GLCMs was utilized. GRLM parameters were calculated from four GRLMs corresponding to four angles (0°, 45°, 90°, 135°), and the mean value of each feature over the four GRLMs was utilized. DWT parameters were calculated for two layers and three directions (horizontal, vertical, diagonal) to produce low and high frequency components. For example, harr_L represented the low frequency component using harr wavelet, and harr_DH2 represented the diagonal high frequency component of the second layer using harr wavelet.

**Table 1:** Details of extracted texture features.

Methods	Texture features	Quantity
**Histogram**	Mean, Variance, Skewness, Kurtosis	4
		
**GLCM**	Autocorrelation, Contrast, Correlation, Cluster Prominence, Cluster Shade, Dissimilarity, Energy, Entropy, Homogeneity, Maximum Probability, Variance, Sum Average, Sum Variance, Sum Entropy, Difference Variance, Difference Entropy, Information Measure of Correlation 1, Information Measure of Correlation 2, Inverse Difference Normalized	19
		
**GRLM**	Short Run Emphasis, Long Run Emphasis, Gray Level Nonuniformity, Run-Length Nonuniformity, Run Percentage, Low Gray Level Run Emphasis, High Gray Level Run Emphasis, Short Run Low Gray Level Emphasis, Short Run High Gray Level Emphasis, Long Run Low Gray Level Emphasis, Long Run High Gray Level Emphasis	11
		
**DWT**	Harr parameters	7
	Deubechies2 parameters	7
	Symlet4 parameters	7
**Total**		55

GLCM, gray level co-occurrence matrix; GRLM, gray level run-length matrix; DWT, discrete wavelet transformation.

### Model Construction and Statistical Analysis

The clinical characteristics and kinetic parameters of the patients were statistically analyzed using SPSS 22.0 (IBM, Corp). Categorical variables included estrogen receptor status, progesterone receptor status, Ki-67 status, histological grade, and histological type, and these variables were compared between HER2-positive and -negative groups using the chi-square test or Fisher’s exact test. For quantitative data including age, maximum tumor diameter, kinetic parameters, and texture features, the independent sample *t*-test was utilized when the data was normally distributed with homogeneous variance, and the Mann-Whitney *U* test was used when the data was not normally distributed. A two-sided *P* value less than 0.05 was considered statistically significant.

The data from 72 patients in the training set were used for feature selection and model construction. Feature selection was performed using MATLAB 2018a. Separately for each kinetic map, Pearson’s correlation analysis was first performed among features in the training set. Highly correlated features with coefficients greater than 0.95 were marked, and the ones with higher correlations with other features were removed. Then, the least absolute shrinkage and selection operator (LASSO) was used to select features with nonzero coefficients among the remaining features by 10-fold cross-validation. After removal, the features were randomly divided into 10 groups. At each feature selection loop, one group of features was chosen as the validation set and the remaining groups were used as the training set. The optimal subset of features for prediction was generated after each loop, and this process was repeated for all ten folds. All selected features were recorded for further analysis.

The multivariate logistic regression analysis using forward stepwise selection was applied with entry of the selected features to establish the prediction model. Spearman’s correlation analysis was performed to evaluate the correlation between texture features contained in the model and HER2 status. The performance of the trained model was assessed through the area under the receiver operating characteristic (ROC) curve (AUC). The sensitivity, specificity, and accuracy were calculated correspondingly. The optimal threshold was chosen according to the maximum Youden index. The established prediction model was further tested on the test set using the same threshold determined on the training set. The corresponding AUC, sensitivity, specificity, and accuracy were also calculated. The above analysis was performed on MedCalc (version 14.10.20, http://www.medcalc.org/).

## Results

### Characteristics of the Study Population

**Figure 2:**
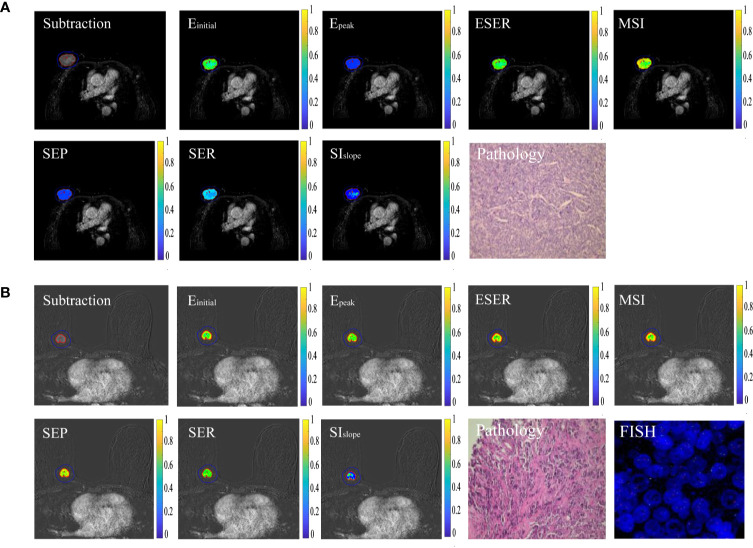
Typical cases of HER2 positivity and negativity. **(A)** Sample images of HER2 positivity, including lesion segmentation, seven semiquantitative DCE maps, and corresponding pathological results. **(B)** Sample images of HER2 negativity.

A total of 102 patients (51.60 ± 10.10 years) were included in this study. The detailed clinical and histopathological characteristics between HER2-positive and -negative groups are listed in **Table 2**. There was no statistical difference between the two groups with respect to age (*P* = 0.57), maximum tumor diameter (*P* = 0.26), histological grade (*P* = 0.17), or histological type (*P* = 0.91). The two groups showed significant differences in terms of estrogen receptor status (*P* < 0.01), progesterone receptor status (*P* = 0.02), and Ki-67 status (*P* = 0.04). **Figure 2** shows two randomly selected cases used to display the results of lesion segmentation along with seven semiquantitative DCE maps and corresponding pathological results.

**Table 2:** Clinical and histopathological characteristics of all patients.

Characteristics	HER2 status		*P*-value
	Positive (n = 48)	Negative (n = 54)	
**Age (mean ± SD)**	50.96 ± 10.59	52.09 ± 9.69	0.57^a^
**Maximum tumor diameter (mm)**	20.79 ± 5.13	19.69 ± 4.79	0.26^a^
**Estrogen receptor status**			**<0.01** ^b^
Positive	26 (54.20%)	43 (79.60%)	
Negative	22 (45.80%)	11 (20.40%)	
**Progesterone receptor status**			**0.02** ^b^
Positive	21 (43.80%)	36 (66.70%)	
Negative	27 (56.20%)	18 (33.30%)	
**Ki-67 status**			**0.04** ^c^
≥14%	44 (91.70%)	40 (74.10%)	
<14%	4 (8.30%)	14 (25.90%)	
**Histological grade**			0.17^c^
I	0	3 (5.60%)	
II	33 (68.80%)	39 (72.20%)	
III	15 (31.20%)	12 (22.20%)	
**Histological type**			0.91^c^
Invasive carcinoma of no special type	45 (93.80%)	50 (92.60%)	
Ductal carcinoma in situ	3 (6.20%)	2 (3.70%)	
Invasive lobular carcinoma	0	1 (1.75%)	
Invasive micropapillary carcinoma	0	1 (1.75%)	

a Variables were tested using the independent sample t-test.

b Variables were tested using the χ^2^ test.

c Variables were tested using Fisher’s exact test.

The bold P-values are considered statistically significant.

### Performance of the Prediction Model

The comparison results of the average value of seven kinetic parameters in the lesion area between HER2-positive and -negative groups is provided in **Table 3**. There were no significant differences in the average value of seven kinetic parameters between the two groups. **Table 4** presents the logistic regression models obtained from the training set. **Table 5** shows comparison results of texture features included in models in the training set between HER2-positive and -negative groups. Short Run Emphasis derived from E_initial_, ESER, MSI, and SER maps were significantly different between HER2-positive and -negative patients (*P* < 0.01, < 0.01, < 0.01, and < 0.01, respectively). Contrast (*P* < 0.01) and harr_HH2 (*P* < 0.01) from E_peak_ maps, autocorrelation (*P* < 0.01) from SEP maps, and gray level nonuniformity (*P* < 0.01) from SI_slope_ were also significantly different between the two groups.

**Table 3:** Comparison results of the average value of seven kinetic parameters from the lesion area.

Parameters	HER2 Positive	HER2 Negative	*P*-value
**E_initial_**	185.57 ± 10.95	173.52 ± 10.08	0.42
**E_peak_**	269.21 ± 104.98	276.41 ± 87.37	0.71
**ESER**	80.80 ± 13.94	63.41 ± 75.78	0.12
**MSI**	110.45 ± 31.41	106.13 ± 33.13	0.50
**SEP**	227.19 ± 86.98	227.47 ± 82.95	0.99
**SER**	132.47 ± 23.23	131.13 ± 19.68	0.75
**SI_slope_**	7.68 ± 17.26	12.26 ± 16.72	0.18

Variables were tested using the independent sample t-test.

**Table 4:** Logistic regression models.

Parameter maps	Logistic regression model
**E_initial_**	*Y* = 11.52-0.42*Kurtosis-15.89*Short Run Emphasis
**E_peak_**	*Y* = 1.23 + 6.39*Contrast-1.02*harr_HH2
**ESER**	*Y* = 8.33-13.80*Short Run Emphasis
**MSI**	*Y* = 8.53-14.83*Short Run Emphasis
**SEP**	*Y* = -4.46+1.80*Autocorrelation
**SER**	*Y* = 7.90-12.60*Short Run Emphasis
**SI_slope_**	*Y* = -24.22+0.34*harr_DH2+0.24*symlet4_HH1+0.12*Gray Level Nonuniformity-0.01*High Gray Level Run Emphasis-0.08*Mean

**Table 5:** Comparison of texture features included in the logistic regression models in the training set between HER2-positive and -negative groups.

Parameter maps	Texture features	HER2 Positive	HER2 Negative	*P*-value	Correlation with HER2 status (r_s_)
**E_initial_**	Kurtosis	4.53 ± 2.99	5.72 ± 3.28	0.12^a^	-0.30
	Short Run Emphasis	0.55 (0.45-0.62)	0.67 (0.62-0.71)	**<0.01** ^b^	-0.52
					
**E_peak_**	Contrast	0.34 (0.21-0.52)	0.19 (0.16-0.26)	**<0.01** ^b^	0.44
	harr_HH2	2.07 (1.66-2.54)	2.40 (2.15-3.35)	**<0.01** ^b^	-0.50
					
**ESER**	Short Run Emphasis	0.53 (0.43-0.64)	0.68 (0.62-0.73)	**<0.01** ^b^	-0.57
					
**MSI**	Short Run Emphasis	0.49 (0.41-0.60)	0.65 (0.61-0.70)	**<0.01** ^b^	-0.60
					
**SEP**	Autocorrelation	3.14 (2.14-3.61)	1.92 (1.56-2.42)	**<0.01** ^b^	-0.37
					
**SER**	Short Run Emphasis	0.56 (0.47-0.67)	0.69 (0.64-0.74)	**<0.01** ^b^	-0.54
					
**SI_slope_**	harr_DH2	5.01 ± 1.84	4.79 ± 2.07	0.63^a^	0.08
	symlet4_HH1	10.31 ± 6.36	10.20 ± 4.48	0.93^a^	-0.07
	Gray Level Nonuniformity	263.25 (217.54-400.84)	215.25 (209.30-226.33)	**<0.01** ^b^	0.43
	High Gray Level Run Emphasis	1.23E+5 (1.00E+5-1.66E+5)	1.44E+5 (1.27E+5-1.57E+5)	0.10^b^	-0.20
	Mean	9.60 ± 19.63	13.00 ± 17.87	0.44^a^	-0.08

a Variables were tested using the independent sample t-test.

b Variables were tested using the Mann-Whitney U test.

The bold P-values are considered statistically significant.

**Figure 3:**
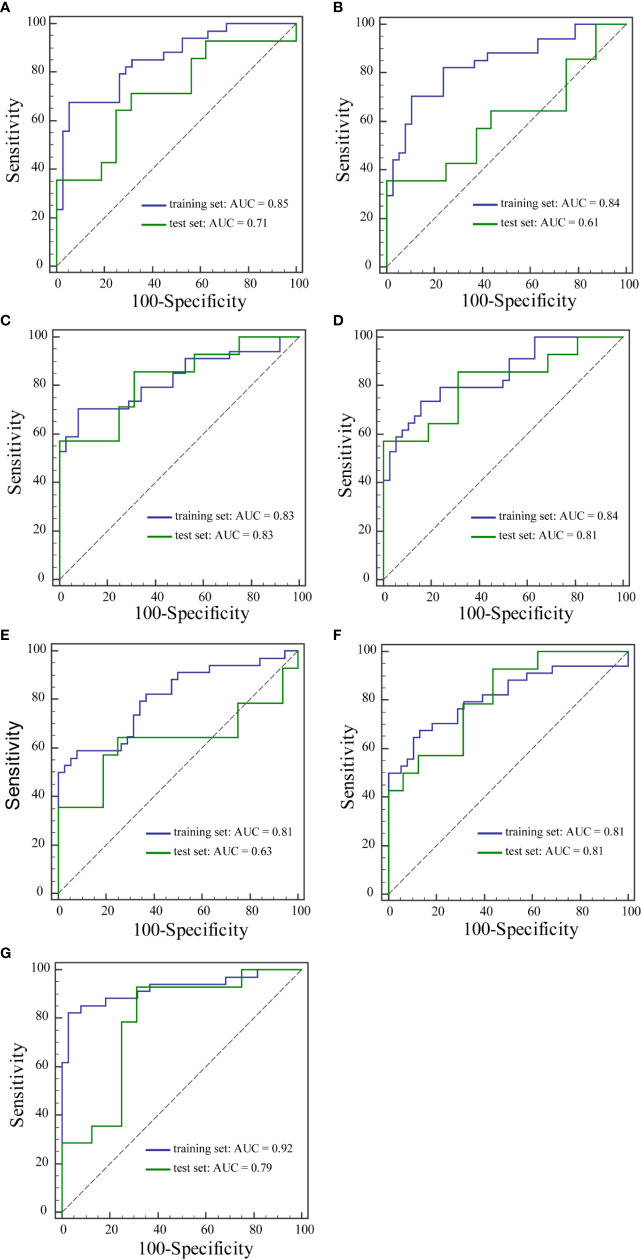
ROC curves of the training set and the test set from Einitial **(A)**, Epeak **(B)**, ESER **(C)**, MSI **(D)**, SEP **(E)**, SER **(F)**, and SIslope **(G)** maps.

The performance of the prediction models is summarized in **Table 6**. Among the seven prediction models, models with features extracted from the ESER map yielded an AUC of 0.83 in the training set [95% confidence interval (CI), 0.72–0.91; sensitivity of 70.59%, specificity of 92.11%, and accuracy of 81.94%], and the highest AUC of 0.83 in the test set (95% CI, 0.64–0.94; sensitivity of 57.14%, specificity of 100.00%, and accuracy of 80.00%). The model with features extracted from the SI_slope_ map yielded the highest AUC of 0.92 in the training set (95% CI, 0.84–0.97; sensitivity of 82.35%, specificity of 97.37%, and accuracy of 90.28%), and an AUC of 0.79 in the test set (95% CI, 0.59–0.91; sensitivity of 92.86%, specificity of 68.75%, and accuracy of 80.00%). The corresponding ROC curves of the models with features extracted from the seven kinetic parameter maps are shown in **Figure 3**.

**Table 6:** Performance of prediction models.

	AUC	95% CI	Sensitivity	Specificity	Accuracy
**E_initial_**					
Training set	0.85	0.75-0.93	67.65%	94.74%	81.94%
Test set	0.71	0.52-0.86	71.43%	68.75%	70.00%
**E_peak_**					
Training set	0.84	0.73-0.91	70.59%	89.47%	80.56%
Test set	0.61	0.42-0.78	35.71%	100.00%	70.00%
**ESER**					
Training set	0.83	0.72-0.91	70.59%	92.11%	81.94%
Test set	0.83	0.64-0.94	57.14%	100.00%	80.00%
**MSI**					
Training set	0.84	0.74-0.92	73.53%	84.21%	79.17%
Test set	0.81	0.63-0.93	57.14%	100.00%	80.00%
**SEP**					
Training set	0.81	0.70-0.89	58.82%	92.11%	76.39%
Test set	0.63	0.44-0.80	64.29%	75.00%	70.00%
**SER**					
Training set	0.81	0.71-0.90	67.65%	86.84%	77.78%
Test set	0.81	0.63-0.93	92.86%	56.25%	73.33%
**SI_slope_**					
Training set	0.92	0.84-0.97	82.35%	97.37%	90.28%
Test set	0.79	0.59-0.91	92.86%	68.75%	80.00%

AUC, area under the receiver operating characteristic curve; CI, confidence interval.

## Discussion

In this study, the correlation between texture features and HER2 status in breast cancer was investigated using a texture analysis of seven semiquantitative kinetic parameter maps based on breast DCE-MRI. The results demonstrated that texture analysis based on DCE-MRI images has the potential to discriminate HER2 status in breast cancer. HER2 is a cell surface receptor expressed in normal breast cells that controls growth, division, and repair of breast cells (26). HER2-positive breast cancer is considered an aggressive disease, because the amplification of the HER2 gene results in an abnormally high amount of HER2 gene expression and HER2 proteins per cancer cell. Therefore, HER2-positive cancers promote the rapid growth and division of cancer cells, and the prognosis is generally poor (27). Trastuzumab treatment can be beneficial for breast cancer with HER2 amplification and overexpression, and therefore the HER2 status serves as a guide for treatment and is a crucial indicator of prognosis (28).

In recent years, there have been some studies on the association between radiomic features and HER2 status (22–24, 26, 29–31). Several studies investigated the relationship between HER2 status and radiomic features in gastric cancer and colorectal cancer (29–31). Li et al. (29) built and validated a CT-based radiomics nomogram for HER2 status prediction, which showed good performance. Zhou et al. (22) reported that mammography radiomics features can effectively diagnose HER2 status of patients with breast cancer, most notably with a model built using a combination of features from cranial caudal and mediolateral oblique views. One study indicated that radiomics features from multidetector computed tomography images were associated with HER2 status in patients with breast cancer (23). Another study developed support vector machine models based on radiomic features from fat-suppressed T2-weighted images and DCE-MRI and, using a combination of these features, noninvasively evaluated the HER2 status of patients with breast cancer. The model based on the combination of fat-suppressed T2-weighted images and DCE-MRI exhibited the best performance for predicting the HER2 status of patients with an AUC of 0.86 and an accuracy of 79.5% in the primary cohort, and an AUC of 0.81 and an accuracy of 78.3% in the validation cohort (24). However, no previous studies have explored the relationship between HER2 status in breast cancer and radiomic features derived from semiquantitative kinetic parameter maps based on breast DCE-MRI.

In the present study, the association between texture features from semiquantitative kinetic parameter maps and HER2 status in breast cancer was investigated. Fifty-five texture features were extracted from each of the seven semiquantitative kinetic parameter maps for each patient. Logistic regression models using forward stepwise selection were developed and validated to predict different HER2 status in breast cancer. Among the seven prediction models based on texture features from E_initial_, E_peak_, ESER, MSI, SEP, SER, and SI_slope_ maps, two of the prediction models showed relatively good performance. The model built using features from the ESER map yielded an AUC of 0.83 in the training set, and the highest AUC of 0.83 in the test set. The model with features extracted from the SI_slope_ map yielded the highest AUC of 0.92 in the training set, and an AUC of 0.79 in the test set. The texture features selected in the seven models included mean, kurtosis, contrast, autocorrelation, gray level nonuniformity, short run emphasis, high gray level run emphasis, and three DWT features. Contrast represents local variations presented in maps. Autocorrelation detects repetitive patterns of texture elements. Gray level nonuniformity measures the similarity of the gray level throughout the lesion area. Short run emphasis reflects the distribution of short runs. Compared with HER2-negative breast cancer, semiquantitative kinetic parameter maps of HER2-positive breast cancer showed higher contrast, autocorrelation, and gray level nonuniformity, as well as lower short run emphasis in the training set. The manifestation of these features indicated that semiquantitative kinetic parameter maps of HER2-positive breast cancer may show more heterogeneity and higher texture complexity than HER2-negative breast cancer.

Fusco et al. (32) calculated 10 semiquantitative kinetic parameters, maximum signal difference (MSD), time to peak between wash-in and wash-out segments, wash-in slope (WIS), wash-out slope (WOS), wash-in intercept, wash-out intercept, area under the curve of wash-in, area under the curve of wash-out, area under the curve of wash-in and wash-out, and standardized index of shape (SIS) as well as 50 textural features to predict breast cancer therapy response. The results demonstrated that SIS achieved the highest AUC value (0.93), suggesting that the joint feature from semiquantitative parametric maps may obtain the best diagnostic performance. In our study, we evaluated the texture features based on seven independent semiquantitative kinetic parameter maps.

A recent study showed that deforming autoencoder convolutional neural networks based on 3TP (three-channel images representing a given slice at three different time points, uniquely identified by means of the three time points) slices of DCE-MRI could be developed to discriminate malignant from benign lesions, and good diagnostic performance was achieved (33). In comparison, the performance of deep learning features was not investigated in our research, as we focused solely on the feasibility of the texture features from semiquantitative kinetic parameter maps. In addition, MRI scans in our study included more phases for post-contrast acquisitions, based on which of the seven kinetic maps were obtained. To improve the performance of the prediction model, further work should be conducted to develop the classification models by combining texture and deep learning features from kinetic maps of DCE-MRI for preoperative prediction of HER2 status in patients with breast cancer.

This study had several limitations. First, the sample size in this study was relatively small and this may have impeded the generalizability of the findings. Second, our results may not be applicable in all other institutions as our study was performed in a single institution with uniform MRI parameters. Additional studies are needed to increase cohort size and consider various conditions. Third, only the texture features extracted from semiquantitative kinetic parameter maps were used to discriminate different HER2 status in breast cancer in this study. Other clinical and histopathological characteristics such as volume, tumor location, lymph-vascular invasion, and diffusion-weighted imaging radiomics may also be good signatures for distinguishing positive and negative status of hormone receptors in breast cancer (25, 34). Combining texture features and clinical characteristics in models may improve prediction performance. Fourth, the slices with maximum tumor diameter were selected and utilized in our study. Texture analysis was performed on two-dimensional images, and the representation of the entire volume of the tumor may have been limited compared with three-dimensional analysis. Finally, Piantadosi et al. (35) reported a U-shaped deep convolutional neural network that exploited the well-known 3TP approach for the automatic lesion segmentation task, which showed a good result in breast DCE-MRI segmentation. However, our study used the semi-automatic segmentation based on Otsu’s algorithm, which was time-consuming. Thus, in our future research, automatic lesion segmentation will be performed using the U-shaped deep convolutional neural network.

## Conclusion

In conclusion, our results indicated that texture features derived from kinetic parameter maps, calculated based on breast DCE-MRI, have the potential to be used as imaging biomarkers for distinguishing HER2-positive and HER2-negative breast cancer. Further studies with larger sample sizes are necessary to verify the results of this study.

## Data Availability Statement

The original contributions presented in the study are included in the article/supplementary material/Further inquiries can be directed to the corresponding author.

## Ethics Statement

Written informed consent was not obtained from the individual(s) for the publication of any potentially identifiable images or data included in this article.

## Author Contributions

LS performed the experiment and wrote the paper. CL revised the manuscript. JY designed the study. All authors contributed to the article and approved the submitted version.

## Funding

This research was supported by grants from the Research and Development (R&D) Foundation for Major Science and Technology from Shenyang (No. 19-112-4-105), the Big Data Foundation for Health Care from China Medical University (No. HMB201902105), the Natural Fund Guidance Plan from Liaoning (No. 2019-ZD-0743), and the 345 Talent Project from Shengjing Hospital of China Medical University.

## Conflict of Interest

The authors declare that the research was conducted in the absence of any commercial or financial relationships that could be construed as a potential conflict of interest.
